# Digital health interventions and quality of home-based primary care for older adults: A scoping review protocol

**DOI:** 10.3389/fpubh.2022.1022587

**Published:** 2023-01-09

**Authors:** Ísis de Siqueira Silva, Cícera Renata Diniz Vieira Silva, Rayssa Horácio Lopes, Aguinaldo José de Araújo, Renan Cabral de Figueirêdo, Osvaldo de Goes Bay, Luís Velez Lapão, Pedro Bezerra Xavier, Severina Alice da Costa Uchôa

**Affiliations:** ^1^Postgraduate in Collective Health, Federal University of Rio Grande do Norte, Natal, Brazil; ^2^Technical School of Health of Cajazeiras, Teacher Training Center, Universidade Federal de Campina Grande, Cajazeiras, Brazil; ^3^Postgraduate in Family Health, Federal University of Rio Grande do Norte, Natal, Brazil; ^4^Faculty of Health Sciences of Trairi, Federal University of Rio Grande do Norte, Santa Cruz, Brazil; ^5^Global Health and Tropical Medicine, Instituto de Higiene e Medicina Tropical, Universidade Nova de Lisboa, Lisbon, Portugal; ^6^Postgraduate in Collective Health, Federal University of Rio Grande do Norte, Santa Cruz, Brazil; ^7^Public Health Department, Federal University of Rio Grande do Norte, Natal, Brazil

**Keywords:** digital health, telemedicine, home-based primary care, older adults, geriatric care, quality in healthcare, scoping review, digital health interventions

## Abstract

**Introduction:**

The use of digital health interventions has expanded, particularly in home-based primary care (HBPC), following the increase in the older adult population and the need to respond to the higher demand of chronic conditions, weakness and loss of autonomy of this population. There was an even greater demand with COVID-19 and subsequent isolation/social distancing measures for this risk group. The objective of this study is to map and identify the uses and types of digital health interventions and their reported impacts on the quality of HBPC for older adults worldwide.

**Methods and analysis:**

This is a scoping review protocol which will enable a rigorous, transparent and reliable synthesis of knowledge. The review will be developed from the theoretical perspective of Arksey and O'malley, with updates by Levac and Peters and respective collaborators based on the Joanna Briggs Institute manual, and guided by the Preferred Reporting Items for Systematic Reviews and Meta-Analyses Extension for Scoping Reviews (PRISMA-ScR). Data from white literature will be extracted from multidisciplinary health databases such as: the Virtual Health Library, LILACS, MEDLINE/PubMed, Scopus, Web of Science, Cinahl and Embase; while Google Scholar will be used for gray literature. No date limit or language restrictions will be determined. The quantitative data will be analyzed through descriptive statistics and qualitative data through thematic analysis. The results will be submitted to stakeholder consultation for preliminary sharing of the study and will later be disseminated through publication in open access scientific journals, scientific events and academic and community journals. The full scoping review report will present the main impacts, challenges, opportunities and gaps found in publications related to the use of digital technologies in primary home care.

**Discussion:**

The organization of this protocol will increase the methodological rigor, quality, transparency and accuracy of scoping reviews, reducing the risk of bias.

## 1. Introduction

The increase in the older adult population and the subsequent need for health systems to respond to issues of chronic diseases, weaknesses, and loss of autonomy has increased the demand for home-based primary care (HBPC) around the world. HBPC includes care that seeks to adequately meet the social and health needs of people in the residential environment. Actions are offered for promotion, prevention, minimization of disease sequelae, situations of weakness and loss of autonomy, monitoring of chronic diseases, palliative care, and support in activities of daily living. These actions can be technical, offered by health professionals or laypeople, the result of intuition, and support in daily life activities care for older adults and self-care guided by professionals (1–4).

The World Health Organization has been evaluating the challenges of home-based care on the European continent, and the analysis presents several issues, among which include the need for governments to regulate the private sector, and to have policies focused on quality, accessibility, efficiency and equity. In this direction, the analysis warns that the aging population requires appreciation of public funding which is specific to home care within health financing, highlighting the relevance of Primary Health Care (5).

Home care is one of the PHC priorities, especially for those who cannot easily commute to health services (6). Studies about PHC and home care articulation present advantages such as providing users with mechanisms to access longitudinal care and promoting improved quality of care with lower costs due to a stronger relationship between the person and their caregiver (7, 8). Expanding coverage and quality of services are of paramount importance for PHC (9) as a strategy to reorganize health systems in order to guarantee longitudinal and comprehensive care for chronic patients in the territories covered, especially in cases where HBPC is the timeliest form of care (5, 9).

An important additional challenge for the quality of HBPC is the need for complex coordination due to the interdependence of health services, as this coordination can be performed by Primary Healthcare (PHC), hospitals or nursing services (5), with advantages for coordination by PHC (5).

HBPC demand has significantly increased during the COVID-19 pandemic, considering that older adults, carriers of chronic diseases and affected by immunosenescence, are more susceptible to infectious diseases (7). HBPC was used to reduce attendance at emergency services and ensure that chronic medical problems were treated within the home environment to prevent their worsening (10, 11). Faced with the challenges in PHC from COVID-19, the use of Information and Communication Technologies (ICT). Moreover, digital health gained even more prominence due to the operability and versatility of generating information at an opportune time (12–14).

Digital health can be defined as a safe and cost-effective way of using information and communication technologies in health and related areas (15, 16). Its scope includes several informational areas such as artificial intelligence, big data, blockchain, health data, health information systems, infodemics, internet of things, teleconsultations, telemonitoring, e-learning and mHealth (16, 17). Digital health assists healthcare workers in diagnosing, monitoring, and communicating with older adult patients around the world, especially during the COVID-19 pandemic (18). Its use can contribute to strengthen health systems by quickly making reliable and up-to-date health information available (16).

Digital health can be used to accelerate the achievement of global health and wellbeing and to expand older people's access to quality PHC (12–15). Some studies emphasize the possibilities of using digital health in home care among the older adults. A study conducted in Indonesia identified needs and opportunities for enabling the use of cell phones and mobile applications for the health of older adults (18).

On the other hand, there are barriers to the use of digital health, such as the digital divide, the fact that half of the world's population is still offline, and the contrast between developed and developing countries is enormous (19). In addition, older adults with lower socioeconomic status have reduced access to digital resources and may not be able to afford the technology or internet needed to use digital tools (20).

In a preliminary search conducted in November 2022 on MEDLINE/PubMed and Google Scholar using the keywords: Aged; Telemedicine; Digital health; and Primary Health Care, review articles were found that explored the uses and experiences of digital health technologies used in care for older adults (21–25). However, no review studies were found that established an association between digital health, home care for older adults and health quality.

Thus, the objective of this study is identify and map the uses and types of digital health interventions and their impacts on the quality of primary home care for older adults worldwide. The Donabedian model approach will be used for the concept of quality applied to healthcare, as it presents a set of desirable attributes which are called (the seven) pillars of quality: efficacy, effectiveness, efficiency, optimization, acceptability, legitimacy and equity (26, 27). These seven pillars are defined in three dimensions: technical (accuracy in the choice of actions and the way in which they are produced), interpersonal (social and psychological relationships between care providers and users) and organizational (conditions in which services are offered comprehensively and with continuity of care, coverage, coordination of actions, access and accessibility to services).

## 2. Materials and methods

**Figure 1:**
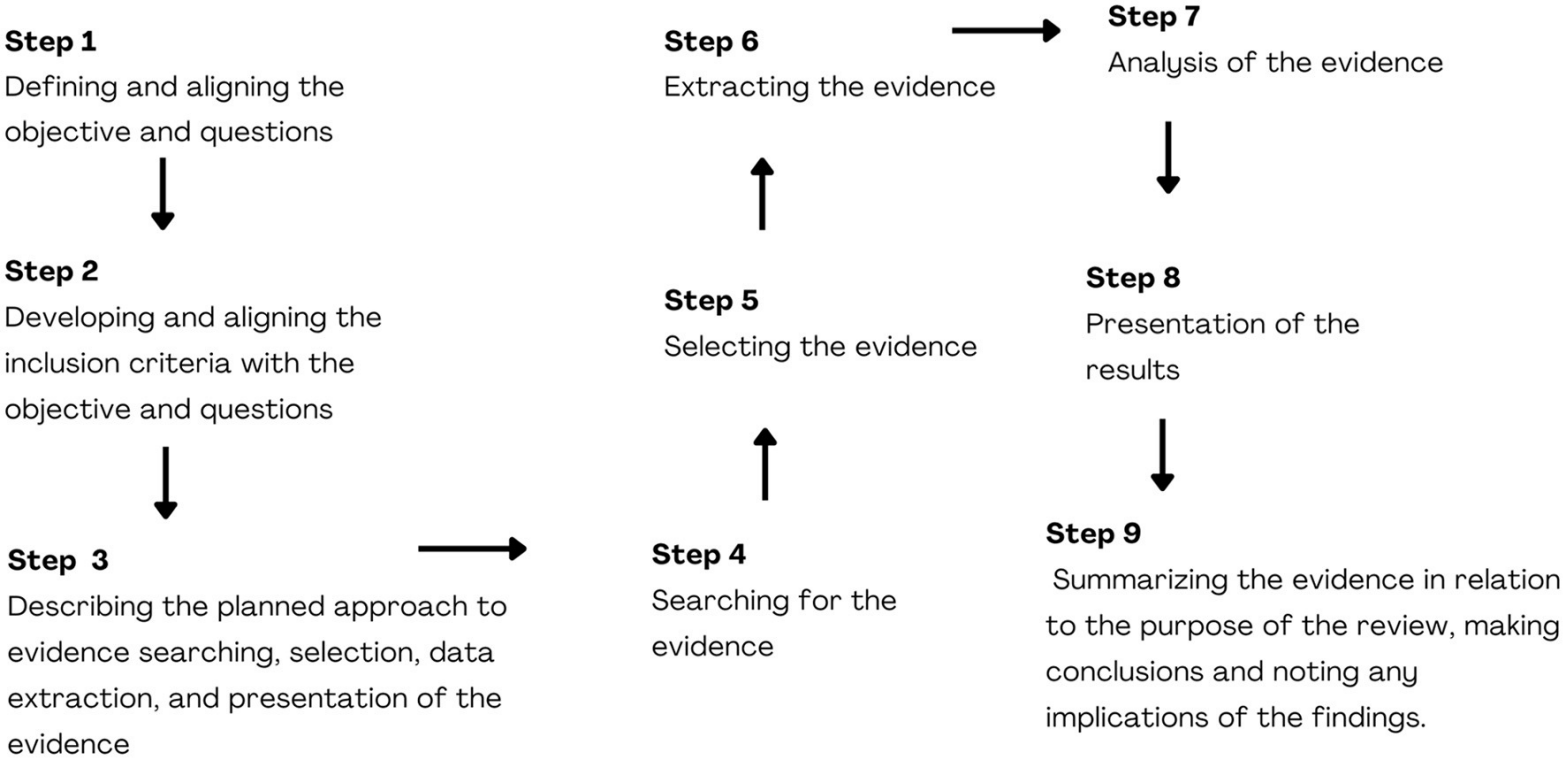
Steps of the scoping review (32). Source: prepared by the authors, 2022.

This study is a scoping review protocol which seeks to answer broader research questions. The study will identify and map emerging evidence on the topic addressed, synthesizing knowledge with rigor, transparency and reliability. It is based on Joanna Briggs Institute (JBI) criteria guided by the theoretical framework of Arksey and O'malley (28), with updates from Levac et al. (29) and Peters et al. (30), as well as by the Preferred Reporting Items for Systematic Reviews and Meta-Analyses Extension for Scoping Reviews (PRISMA-ScR) (31). The protocol was registered in the Open Science Framework (OSF) (https://osf.io/vgkhy). As shown in Figure 1, the nine steps of the Scoping Review include: (1) Defining and aligning the objective/s and question/s; (2) Developing and aligning the inclusion criteria with the objective and questions; (3) Describing the planned approach to evidence searching, selection, data extraction, and presentation of the evidence; (4) Searching for the evidence; (5) Selecting the evidence; (6) Extracting the evidence; (7) Analysis of the evidence; (8) Presentation of the results; (9) Summarizing the evidence in relation to the purpose of the review, making conclusions and noting any implications of the findings (32).

### 2.1. Step 1: Defining and aligning the objective and questions

Objective: Identify and map the uses and types of digital health interventions and their impacts on the quality of primary home care for older people worldwide.

The research questions were formulated through the PCC mnemonic conceptual model—(Population, Concept, Context) (31), as:

**P**: Older adults;

**C**: Digital health interventions;

**C**: Home-based primary care.

The following research questions were prepared by the authors according to the PCC:

1. Which countries use digital health interventions in home-based primary care for older adults?
2. What sort of digital health interventions are used in home-based primary care for older adults?
3. What is the measured impact of digital health interventions on the quality of home-based primary care for older adults?

The key concepts for elaborating the research questions are described in Table 1.

**Table 1:** Key concepts for the study questions.

**Concept**	**Definition**
Older adult	For the World Health Organization (33), the concept of “old age” is multidimensional, and includes the terms chronological (based on the date of birth), biological (related to the capacity of the human body), psychological (related to the psycho-emotional functioning) and social age (related to social roles). For the United Nations (34), the definition of an older adult is related to those who are 60 years old or more, but at the same time they affirm that there is a diversity of older people with different needs, abilities, lifestyles, experiences and preferences which are influenced by age, gender, health, income, education, ethnicity and other factors.
Digital health interventions	The classification of digital health interventions (DHIs) categorizes the different ways in which digital technologies are being used to support health system needs. This classification framework is primarily targeted at public health audiences, and aims to promote an accessible and bridging language for health program planners to articulate functionalities of digital health implementations (16).
HBPC (home-based primary care	This is a model of providing long-term primary care at home, ranging from palliative care, rehabilitation, and disease management to care coordination. The multidisciplinary team has older adults with chronic diseases and physical and cognitive disabilities as its main clientele (35, 36).
Care quality	The quality of care can be defined in three dimensions: technical (accuracy in the choice of actions and the way in which they are produced), interpersonal (social and psychological relationships between care providers and users) and organizational (conditions in which services are offered comprehensively and with continuity of care, coverage, coordination of actions, access and accessibility to services (26).

Source: prepared by the authors, 2022.

### 2.2. Step 2: Developing and aligning the inclusion criteria with the objective and questions

Publications that address the use of digital health interventions in HBPC for older adults will be included, available in full, which answer the study questions.

The following will be included:

a) Primary studies, theoretical and brief communications.
b) Gray literature, including government manuals, expert opinions and brief communications as well as dissertations and theses.

Time filters will not be applied to the searches, as the search strategies will contain descriptors and terms referring to digital health. The search will not be limited by date or language. Duplicate publications, literature reviews, editorials, will be excluded.

### 2.3. Step 3: Describing the planned approach to evidence searching, selection, data extraction, and presentation of the evidence

The following steps will be taken to enhance the identification of documents in white literature and gray literature:

The initial search was conducted in MEDLINE/PubMed using Medical Subject Headings (MeSH) in English to identify main descriptors, synonyms, and keywords included in titles, abstracts, and indexed terms of publications regarding the theme. A similar search was conducted in Portuguese using the Virtual Health Library (VHL) and *Descritores em Ciências da Saúde* (*DeCS*).

Moreover, a librarian improved the search strategy using four controlled vocabularies (DeCS, MeSH terms, Emtree terms; Cinahl headings) to obtain a wide range of multidisciplinary results in different databases. Natural language (non-controlled vocabulary) was also used to increase the sensitivity of the strategy (37).

The search strategy was constructed using the Extraction, Conversion, Combination, Construction, and Use model, which enables developing highly sensitive search strategies by following a set of complementary steps (37).

### 2.4. Step 4: Searching for the evidence

English was used to structure the research strategy, considering that it is the main language used in the scientific environment (38). Table 2 organizes the main descriptors available in the DeCS that started the search strategy carried out by the authors based on the PCC, the standard search strategy is available in Appendix I. The detailed search strategy for all data sources (i.e., white and gray literature) will be attached to the final scoping review.

**Table 2:** Descriptors used according to the PCC Mnemonic.

**Mnemonic**	**Descriptor**	**Synonyms/keywords**
P	aged/elderly/frail elderly	Older adults; Aging; older persons; elderly care; aged people; Older Adult
C	Digital health	eHealth; e-Health; telehealth; Telecare; mHealth; Telerehabilitation; Telehomecare; home telehealth; Home telecare; telemonitoring; telecare monitoring system; telenursing; Digital Health; Digital Health Strategies; Digital Health Strategy; Digital Health Interventions; eHealth Strategies and Policies; Telemedicine
C	Home-based care services	home health services; home monitoring; home health care; home care; home-based primary care; Hospitalization at home; Home-based care; Home healthcare; Home-based primary care

Source: Prepared by the authors, 2022.

#### 2.4.1. Data sources

The data collection will be conducted in the following indicated portals and databases: LILACS; MEDLINE/PubMed; Scopus; Web of Science; Cinahl and Embase. Gray literature will be searched through Google Scholar, Open gray, “Gray Matters: a practical tool for searching health-related gray literature”, ProQuest Dissertations and Theses Global and Preprints for Health Sciences [medRXiv]. The appropriate strategy will be applied to each of them, and the title and abstract of all identified studies will be evaluated and the duplicates removed.

The search strategy was pre-tested on MEDLINE/PubMed for white literature (Appendix II) and Google Scholar for gray literature (Appendix III) to check for the possibility of data collection limitations related to the search strategy.

#### 2.4.2. Additional sources

Reference lists of included studies will be consulted for verification of additional publications. If needed, corresponding authors will be contacted *via* e-mail for additional information.

#### 2.4.3. Pilot test

A pilot test will be carried out with two reviewers (IdSS and AJA) before starting data collection in order to reduce bias, ensure alignment in the selection process and testing the form among some team members to refine it and ensure that all relevant data were captured. The two reviewers will be to evaluate the same random sample of 25 papers, evaluating titles and abstracts in a data source and then select them using eligibility criteria. Afterwards, the team will meet to discuss and to resolution the discrepancies, and make necessary changes to the criteria and definitions. Screening will only begin when 75% or more similarity is achieved (32).

### 2.5. Step 5: Selecting the evidence

The study selection process will be guided by the steps proposed in the Preferred Reporting Items for Systematic Review and Meta-Analyses (PRISMA-ScR) (31) for both white and gray literature, which are: (1) identification; (2) screening; (3) eligibility; and (4) inclusion, which will be presented in detail in the review selection diagram.

The selection process of publications belonging to the gray literature will follow the guidelines recommended by Godin et al. (39), with specific strategies for searches on Google Scholar and Preprints repositories. Combinations of the following groups of search terms will be used: Aged OR elderly OR “middle age” OR “old people” OR “very elderly” AND Digital Health OR Telemedicine OR teleconsultation OR “electronic consultation” OR “remote consultation” OR telehealth “home health care” OR “home care”. The search terms and the number of results retrieved for each gray literature search strategy will be recorded and will follow the other proposed selection steps. The results from Google Scholar will be sorted by relevance and the first hundred will be included in the screening (39).

Identified studies will be grouped in the Endnote reference manager and duplicates removed. The Rayyan software program will be used in the evaluation of studies by titles and abstracts to assist in blinding the reviewers (40) and any differences between the two reviewers (IdSS and AJA) will be discussed with a third reviewer (SACU). Studies selected by title and abstract will be retrieved in full and exported to a database in the Microsoft Excel^®^ program. After reading the full text and building the final review sample, data will be extracted by the two independent reviewers, highlighting all reasons for exclusion when necessary and the entire selection process, eligibility, inclusion and reasons for exclusions will be presented in a specific flowchart (31).

### 2.6. Step 6: Extracting the evidence

Data will be extracted according to Appendix IV and included if they align with the objectives and research questions of the scoping review. Data related to the included studies will be extracted by two independent reviewers to reduce the chance of errors and biases using a data extraction form elaborated by authors.

The following items will be extracted from the studies: Type of literature, Publication title, authors, Year of publication, Country, Language. To white literature it will be identified Study design, Study population, Study objective, Research question, Participants, Main results. For both white and gray literature will be extracted type and health situation of digital health interventions used, care actions and its agent and coordination ability to use digital tools, Availability of Digital health interventions and other impacts of using digital health interventions on the quality of home-based primary care.

The instrument can receive updates during the research to obtain a deeper understanding of the theme, as, according to Peters et al. (30).

### 2.7. Step 7: Analysis of the evidence

Descriptive statistics (absolute and percentage frequencies) will be used to analyze quantitative data with the help of the Microsoft Excel^®^ program. Qualitative data analysis will be guided by thematic analysis (41).

This step will be divided into three others, according to Levac (29), namely: (1) data analysis; (2) exposure of results linked to research questions; and (3) interpreting the implications of the results for other research and services.

A map of identified countries that use digital health interventions in HBPC for older adults will be developed using the GeoDa version 1.20 software program (Center for Spatial Data Science, Chicago, IL, USA).

All results will be discussed with the relevant literature. The evidence synthesis will be presented in a descriptive format through tables, diagrams, and thematic maps to better visualize the results found. A narrative summary will follow the mapped data, and report how the results relate to the review objective and questions.

### 2.8. Step 8: Presentation of the results

The final report guided by the PRISMA-ScR (31) will include the results in flowcharts, charts, or figures, and will be presented to a group of stakeholders with experience in digital health. The stakeholder analyses are used throughout the entire planning process of health innovations, more frequently for policies and services and delivery methods (42), and it will be useful for preliminary sharing and suggestion of dissemination of results. The objectives of this strategy, recommended by Levac et al., will be the preliminary sharing of study findings, being considered a mechanism for knowledge transfer and exchange, as well as to develop effective dissemination strategies and ideas for future studies and encourage the search for new evidence or field of research not present in the review (29). In this step, the identification of interested parties will be carried out; the differentiation or categorization of stakeholders based on some attributes, such as power, position, level of interest, possible contributions; and investigating stakeholder relationships with the topic of study (42, 43).

In this protocol, the sample of stakeholders will be intentionally listed through the snowball technique with 9 (nine) stakeholders: researcher (3), health professional (3) and digital professional (3) all with experience in digital health aimed at home-based care. The first included will be identified by the study researchers, who will successively indicate the others.

The procedure will include sending an individual invitation to candidates for research participants, explaining the purpose of their participation and, if they accept, they will sign the Free and Informed Consent Form. Preliminary results and informed consent will be included in an electronic form and sent to stakeholders *via* e-mail. Stakeholders will not be identified, and authors will request the appreciation of dissemination, sharing of results of the review and of the database of publications as well as about possible new fields or evidence for researchers, managers, caregivers and older adults.

### 2.9. Step 9: Summary of evidence, conclusions, implications of findings

The main results will be summarized (including an overview of the concepts, themes and types of evidence available), the research questions and the objective should be answered based on the results found. Expectations about the implications of the findings on digital health interventions and their relevance to the home-based care of older adults will be presented.

## 3. Ethics and dissemination of the results

The study does not directly involve patients, but the stakeholder consultation was approved by the Research Ethics Committee of the Onofre Lopes University Hospital/Federal University of Rio Grande do Norte CAEE 54853921.0.0000.5292. The results will be presented at scientific conferences, events with stakeholders and submitted for open-access publication in a peer-reviewed journal.

## 4. Discussion

This protocol was developed by researchers trained in this type of research and following the methodological criteria suggested by Arksey and O'Malley (28), Levac et al. (29), and JBI (32) guided by the PRISMA-ScR (31). The organization of this protocol will increase the methodological rigor, quality, transparency and accuracy of scoping reviews, reducing the risk of bias. Scoping review protocols contribute to an increasing need to synthesize and summarize research following a reproducible design, implementation and reporting method (44).

The scoping review will be able to present the convergence of two emerging themes, namely, digital health, which offers an opportunity to address health system challenges, improve coverage and maintain the quality of service (45) and home primary care for older adults who demand continuous and sustainable long-term care (7).

## 5. Strengths and limitations

Thus, the main contribution of this study is the elaboration of a protocol with methodological rigor, which will be guide the development of a scope review in the future. The methodological rigor adopted in this protocol, as well as the training and experience of the researchers, will ensure quality and transparency for the development of the scoping review. In addition, this is the first study to propose mapping the use and type of digital health interventions used, and their impacts on the quality of care for older adults. One of the most relevant aspects of the methodology is the inclusion of stakeholder consultation as a way of indicating future strategies for dissemination and applicability of the review results so that they can be more accessible to other researchers, managers, caregivers from different countries.

However, two limitations in the search strategy can be highlighted. The first is that the definition of Digital Health is recent (2020) and evolving. If there are changes in WHO definitions of digital health by the study selection stage, the terms will be updated. The second is the structuring of the search strategy in English which may not include publications from the gray literature in the native language of some countries. Thus, the search strategy may be adapted to Portuguese, Spanish, and French to extend the reach and software will be used to translate these publications into the aforementioned languages. Therefore, we constructed the search in a manner that increased comprehensiveness to minimize the effect of these limitations.

## 6. Conclusion

The present protocol has methodological rigor and is proposed to guide a scoping review that will map and identify the uses and types of health interventions and their impacts on the digital quality of HBPC for older adults worldwide.

The future Scoping Review will provide a reliable source of evidence for managers, digital tool developers and future research to guide the use of digital health interventions in the practice of HBPC for older people, and its results may guide discussions for the elaboration of upcoming healthcare policies and guidelines. Older adults, families, society, caregivers and health professionals will be able to consult the results, identify and decide which digital health intervention best suits their reality, and respond to the demands of care for the elderly, in addition to knowing the impacts of digital health in the HBPC, guided by a scientific study developed with rigor and seriousness.

Results of this review will create socialization with stakeholders and be published in peer-reviewed open-access journals, favoring dissemination of knowledge with the scientific community. Changes in this protocol will be appropriately reported in the final publication, including dates and justifications.

## Author contributions

SU proposed the study and coordinated the elaboration of the protocol. ÍS developed the protocol. ÍS, CS, RL, OB, AA, RF, LL, and SU participated in the discussion of the theoretical and methodological aspects of the study. CS, ÍS, and RL conducted the pilot searches to substantiate the search strategy. PX was in charge of implementing the reviewers' considerations and updating the article and critically reviewed the content. All authors reviewed the protocol and approved its final version for publication. All authors contributed to the article and approved the submitted version.
