# Supplementary material for: Digital health interventions and quality of home-based primary care for older adults: A scoping review protocol
Source: Front Public Health. 2023 Jan 9;10:1022587. doi: 10.3389/fpubh.2022.1022587 (PMC9870288; doi:10.3389/fpubh.2022.1022587)
Supplement: Supplementary file 1 [file Data_Sheet_1.PDF]

APPENDIX I: Standard review search strategy.

Population	Concept	Context
aged OR elderly OR "aged patient" OR "aged people" OR "aged person" OR "aged subject" OR "elderly patient" OR "elderly people" OR "elderly person" OR "elderly subject" OR "senior citizen" OR senium OR "middle aged" OR "middle age" OR "old person" OR "old people" OR "very elderly" OR centenarian OR centenarians OR nonagenarian OR nonagenarians OR octogenarian OR octogenarians OR "very old" OR "oldest old" OR "aged 60" OR "aged 70" OR "aged 80" OR "aged 90" OR "aged 100" OR "60 years" OR "70 years" OR "80 years" OR "90 years" OR "100 years"	Telemedicine OR "m-Health" OR "Mobile Health" OR "mobile healthcare" OR mHealth OR eHealth OR "tele medicine" OR teleconsultation OR "long distance consultation" OR "tele-consultation" OR "telephone consultation" OR "electronic consultation" OR "e-consultation" OR econsultation OR "remote consultation" OR teleconsultations OR telehealth OR "e-health" OR "tele-health" OR telediagnosis OR "remote diagnoses" OR "remote diagnosis" OR "remote diagnostics" OR "tele-diagnosis" OR telediagnoses OR telediagnostics OR telemonitoring OR "distant monitoring" OR "distant patient monitoring" OR "remote monitoring" OR "remote patient monitoring" OR "tele monitoring" OR "video consultation" OR "telemedicine video-consultation" OR videoconsultation OR telepharmacy OR "tele-pharmacy" OR telenursing OR "tele-nursing" OR "digital health" OR "digital-health"	"home care services" OR "home care service" OR "domiciliary care" OR "home health care" OR "home care" OR "domestic health care" OR "domestic healthcare" OR "domiciliary health care" OR "domiciliary healthcare" OR "home care agencies" OR "home care program" OR "home care programme" OR "home care service" OR "home health nursing" OR "home health care nursing" OR "home healthcare" OR "home help" OR "home nursing" OR "home service" OR "home treatment" OR homecare OR "homemaker service" OR "homemaker services"

Search strategy:

(aged OR elderly OR "aged patient" OR "aged people" OR "aged person" OR "aged subject" OR "elderly patient" OR "elderly people" OR "elderly person" OR "elderly subject" OR "senior citizen" OR "middle aged" OR "middle age" OR "old person" OR "old people" OR "very elderly" OR centenarian OR centenarians OR nonagenarian OR nonagenarians OR octogenarian OR octogenarians OR "very old" OR "oldest old" OR "aged 60" OR "aged 70" OR "aged 80" OR "aged 90" OR "aged 100" OR "60 years" OR "70 years" OR "80 years" OR "90 years" OR "100 years") AND (Telemedicine OR "m-Health" OR "Mobile Health" OR "mobile healthcare" OR mHealth OR eHealth OR "tele medicine" OR teleconsultation OR "long distance consultation" OR "tele-consultation" OR "telephone consultation" OR "electronic consultation" OR "e-consultation" OR econsultation OR "remote consultation" OR teleconsultations OR telehealth OR "e-health" OR "tele-health" OR telediagnosis OR "remote diagnoses" OR "remote diagnosis" OR "remote diagnostics" OR "tele-diagnosis" OR telediagnoses OR telediagnostics OR telemonitoring OR "distant monitoring" OR "distant patient monitoring" OR "remote monitoring" OR "remote patient monitoring" OR "tele monitoring" OR "video consultation" OR "telemedicine video-consultation" OR videoconsultation OR telepharmacy OR "tele-pharmacy" OR telenursing OR "tele-nursing" OR "digital health" OR "digital-health") AND ("home care services" OR "home care service" OR "domiciliary care" OR "home health care" OR "home care" OR "domestic health care" OR "domestic healthcare" OR "domiciliary health care" OR "domiciliary healthcare" OR "home care agencies" OR "home care program" OR "home care programme" OR "home care service" OR "home health nursing" OR "home health care nursing" OR "home healthcare" OR "home help" OR "home nursing" OR "home service" OR "home treatment" OR homecare OR "homemaker service" OR "homemaker services")

APPENDIX II – Search Strategy PubMed

* Search performed in the PubMed database on July 21, 2022.

Search	Terms	Records retrieved
#1	Search: aged[Title/Abstract] OR elderly[Title/Abstract] OR "aged patient"[Title/Abstract] OR "aged people"[Title/Abstract] OR "aged person"[Title/Abstract] OR "aged subject"[Title/Abstract] OR "elderly patient"[Title/Abstract] OR "elderly people"[Title/Abstract] OR "elderly person"[Title/Abstract] OR "elderly subject"[Title/Abstract] OR "senior citizen"[Title/Abstract] OR senium[Title/Abstract] OR "middle aged"[Title/Abstract] OR "middle age"[Title/Abstract] OR "old person"[Title/Abstract] OR "old people"[Title/Abstract] OR "very elderly"[Title/Abstract] OR centenarian[Title/Abstract] OR centenarians[Title/Abstract] OR nonagenarian[Title/Abstract] OR nonagenarians[Title/Abstract] OR octogenarian[Title/Abstract] OR octogenarians[Title/Abstract] OR "very old"[Title/Abstract] OR "oldest old"[Title/Abstract] OR "aged 60"[Title/Abstract] OR "aged 70"[Title/Abstract] OR "aged 80"[Title/Abstract] OR "aged 90"[Title/Abstract] OR "aged 100"[Title/Abstract] OR "60 years"[Title/Abstract] OR "70 years"[Title/Abstract] OR "80 years"[Title/Abstract] OR "90 years"[Title/Abstract] OR "100 years"[Title/Abstract]	1,006,503
#2	Search: Telemedicine[Title/Abstract] OR "m-Health"[Title/Abstract] OR "Mobile Health"[Title/Abstract] OR "mobile healthcare"[Title/Abstract] OR mHealth[Title/Abstract] OR eHealth[Title/Abstract] OR "tele medicine"[Title/Abstract] OR teleconsultation[Title/Abstract] OR "long distance consultation"[Title/Abstract] OR "tele-consultation"[Title/Abstract] OR "telephone consultation"[Title/Abstract] OR "electronic consultation"[Title/Abstract] OR "e-consultation"[Title/Abstract] OR econsultation[Title/Abstract] OR "remote consultation"[Title/Abstract] OR teleconsultations[Title/Abstract] OR telehealth[Title/Abstract] OR "e-health"[Title/Abstract] OR "tele-health"[Title/Abstract] OR telediagnosis[Title/Abstract] OR "remote diagnoses"[Title/Abstract] OR "remote diagnosis"[Title/Abstract] OR "remote diagnostics"[Title/Abstract] OR "tele- diagnosis"[Title/Abstract] OR telediagnoses[Title/Abstract] OR telediagnostics[Title/Abstract] OR telemonitoring[Title/Abstract] OR "distant monitoring"[Title/Abstract] OR "distant patient monitoring"[Title/Abstract] OR "remote monitoring"[Title/Abstract] OR "remote patient monitoring"[Title/Abstract] OR "tele monitoring"[Title/Abstract] OR "video consultation"[Title/Abstract] OR "telemedicine video-consultation"[Title/Abstract] OR videoconsultation[Title/Abstract] OR telepharmacy[Title/Abstract] OR "tele-pharmacy"[Title/Abstract] OR telenursing[Title/Abstract] OR "tele-nursing"[Title/Abstract] OR "digital health"[Title/Abstract] OR "digital-health"[Title/Abstract]	56,099
#3	Search: "home care services"[Title/Abstract] OR "home care service"[Title/Abstract] OR "domiciliary care"[Title/Abstract] OR "home health care"[Title/Abstract] OR "home care"[Title/Abstract] OR "domestic health care"[Title/Abstract] OR "domestic healthcare"[Title/Abstract] OR "domiciliary health care"[Title/Abstract] OR "domiciliary healthcare"[Title/Abstract] OR "home care agencies"[Title/Abstract] OR "home care program"[Title/Abstract] OR "home care programme"[Title/Abstract] OR "home health nursing"[Title/Abstract] OR "home health care nursing"[Title/Abstract] OR "home healthcare"[Title/Abstract] OR "home help"[Title/Abstract] OR "home nursing"[Title/Abstract] OR "home service"[Title/Abstract] OR "home treatment"[Title/Abstract] OR homecare[Title/Abstract] OR "homemaker service"[Title/Abstract] OR "homemaker services"[Title/Abstract]	29,451
	**Search** #1 AND #2 AND #3	126
#4	Search: aged[MeSH Terms]) OR (elderly[MeSH Terms])) OR (middle	1,156

	aged[MeSH Terms])) AND (telemedicine[MeSH Terms])) OR ("remote consultation"[MeSH Terms])) OR ("digital health"[MeSH Terms])) AND ("home care services"[MeSH Terms])) OR ("home care"[MeSH Terms])) OR ("home health nursing"[MeSH Terms])	
	**Search** #1 AND #2 AND #3 OR #4	1,248

APPENDIX III – Search Strategy for gray literature in the Google Scholar

* Search performed in Google Scholar on July 20, 2022.

#	Search	Results	Results screened	New potentially relevant records*
1	Aged AND Telemedicine AND home care services	~103.000	100	n
2	elderly OR "aged patient" OR "aged people" AND "m-Health" OR "Mobile Health" OR "mobile healthcare" OR mHealth OR eHealth AND "home care service" OR "domiciliary care" OR "home health care"	~3.570	100	n
3	aged OR elderly AND Telemedicine OR "Digital health" AND "home care"	~19.200	100	n
4	aged AND telemedicine AND "home care"	~8.950	100	n
5	elderly AND telehealth AND "home care"	~10.300	100	n
6	aged AND telemedicine AND "home-based primary care"	~1.050	100	n

APPENDIX IV – Standard Data Collection Instrument

**Study characteristics**	
Study title	
Main author	
Year of publication	
Study country	
Language	
Study design	

Study population	
Study objective	
Research question	
Participants	
Main results	
**Research question data**	
Type of digital health interventions used	
Identify the main uses of digital health interventions used worldwide in home-based primary care for older adults	
Characterization of the older adult’s dependence level	
Assess the impact of using digital health interventions on the quality of home-based primary care	
Agent responsible for care actions	
Availability of Digital health interventions by PHC	
Older adult’s health condition	
Ability to use digital tools	
How the healthcare system is organized	
